# Neuroimmune Support of Neuronal Regeneration and Neuroplasticity following Cerebral Ischemia in Juvenile Mice

**DOI:** 10.3390/brainsci13091337

**Published:** 2023-09-17

**Authors:** Ricaurte A. Marquez-Ortiz, Vesna Tesic, Daniel R. Hernandez, Bilkis Akhter, Nibedita Aich, Porter M. Boudreaux, Garrett A. Clemons, Celeste Yin-Chieh Wu, Hung Wen Lin, Krista M. Rodgers

**Affiliations:** 1 Department of Cellular Biology and Anatomy, Louisiana State University, Health Sciences Center, Shreveport, LA 70803, USAbilkis.akhter@lsuhs.edu (B.A.);; 2 Department of Neurology, Louisiana State University, Health Sciences Center, Shreveport, LA 70803, USA; 3 Department of Pharmacology, Toxicology, and Neuroscience, Louisiana State University, Health Sciences Center, Shreveport, LA 70803, USA

**Keywords:** cerebral ischemia, neural regeneration, neurogenesis, synaptic plasticity, functional recovery, neuroimmune signaling

## Abstract

Ischemic damage to the brain and loss of neurons contribute to functional disabilities in many stroke survivors. Recovery of neuroplasticity is critical to restoration of function and improved quality of life. Stroke and neurological deficits occur in both adults and children, and yet it is well documented that the developing brain has remarkable plasticity which promotes increased post-ischemic functional recovery compared with adults. However, the mechanisms underlying post-stroke recovery in the young brain have not been fully explored. We observed opposing responses to experimental cerebral ischemia in juvenile and adult mice, with substantial neural regeneration and enhanced neuroplasticity detected in the juvenile brain that was not found in adults. We demonstrate strikingly different stroke-induced neuroimmune responses that are deleterious in adults and protective in juveniles, supporting neural regeneration and plasticity. Understanding age-related differences in neuronal repair and regeneration, restoration of neural network function, and neuroimmune signaling in the stroke-injured brain may offer new insights for the development of novel therapeutic strategies for stroke rehabilitation.

## 1. Introduction

Stroke is a destructive neurological disease that is one of the greatest contributors to global disability, and millions of stroke survivors live with life-changing neurological consequences [1,2]. According to recent Global Burden of Disease (GBD) estimates, stroke is the second leading cause of death and third leading cause of disability worldwide, and high morbidity results in up to 50% of stroke survivors being chronically disabled [3,4]. While reports have shown that children have increased resiliency and a longer window for recovery, compared to stroke in the adult population [5,6,7], stroke in pediatric patients can often be more devastating than in adults and the elderly due to poorly understood risk factors and delays in diagnosis which lead to worsened stroke outcomes [8,9,10]. Pediatric stroke remains one of the leading causes of death in children [11] and often results in lifelong neurological disabilities [12]. Yet, little is known about optimal therapies for pediatric stroke survivors or the mechanisms involved in pediatric post-stroke recovery [13]. This creates an urgent need for new targets in stroke rehabilitation that can improve the neurological consequences of stroke in pediatric and adult/aging populations. As neurons in stroke-damaged brain regions die, regenerative responses are initiated that promote neural reorganization, and the reorganization of surviving stroke-damaged brain regions has been found to improve stroke recovery [14,15]. Neurogenesis is a process involving the production of new neurons from neural progenitor cells, which could directly contribute to neural reorganization. New neurons could promote endogenous neural reorganization through synaptic integration into stroke-damaged neural networks, restoring function through new connections that enhance neuroplasticity and subsequently improve post-stroke recovery. 

Neurogenesis is stimulated in both the rodent and human brain in response to stroke [16,17,18]. Following stroke, there is a rapid proliferation and migration of neural progenitor cells, yet most of these cells do not survive past 4 weeks in adult mice [19,20]. However, neurogenesis research has primarily focused on neonatal, perinatal, and adult rodents. This has yielded mixed results because adult neurogenesis differs from developmental neurogenesis, in which the immature brain is still undergoing developmental processes that are limited in mature neurons [21]. Juvenile mice have a fully developed, mature brain that is ideal for studying neurogenesis because it is no longer undergoing developmental processes like those found in neonates and perinates. We have previously reported a robust survival of newly generated neurons at 30 d post-ischemia in the juvenile brain, which was accompanied with improved motor recovery and limb use [22]. Our findings in adults were similar to the existing literature, with low survival rates of newborn neurons and an absence of motor recovery after stroke. Although the early proliferation and migration of immature neurons did not result in brain repair or functional recovery in adults, it signaled an attempt for the brain to repair itself. The survival and maturation of newly generated neurons in juveniles demonstrate the remarkable regenerative capacity of the brain, and investigating the mechanisms that support juvenile neurogenesis could lead to discoveries that promote newborn neuron survival in adults and the elderly. 

The mechanisms of juvenile neurogenesis are not yet understood; however, glial cells have been found to play an important role in every stage of neurogenesis and could provide critical support for juvenile neurogenesis following stroke. Microglia modulate neurogenesis through the secretion of different soluble factors (e.g., cytokines, chemokines, trophic factors, etc.) which affect proliferation, differentiation, and survival of newborn neurons [23] as well as axonal and dendritic growth, synapse formation, and synaptic plasticity [24]. Stroke triggers the activation of innate immunity through danger signaling from damaged and dying cells, releasing endogenous molecules known as damage-associated molecular patterns, which are potent mediators of inflammation that play a fundamental role in activating antigen-presenting cells engaged in host defense [25,26,27]. Microglia, along with astrocytes, are the primary cells involved in the immune response to stroke [28]. It is well established that microglia are rapidly activated during the innate immune response to brain injury, leading to the expression of high levels of proinflammatory cytokines, which play a role in the more delayed conversion of astrocytes into a reactive state [29]. Early glial cell activation is involved in the regulation of cytotoxicity, repair, regeneration, immunosuppression, and other critical aspects of the neuroimmune response to stroke [30]. This is largely due to diverse activation states in microglia, including proinflammatory (cytotoxic and damaging) and anti-inflammatory phenotypes that promote repair and regeneration [31]. Studies have found that during the acute phase of stroke in adults, the primary microglial phenotype is proinflammatory and detrimental to ischemic outcomes [32,33]. However, the influence of stroke-induced neuroimmune responses in the young brain is understudied and poorly understood. 

During the acute phase of stroke in adults, the brain not only activates resident microglia, but also recruits myeloid cells and neutrophils from the periphery [34]. While the immediate response to stroke is primarily mediated by microglia, initial infiltrating monocytes have been reported to be protective [35]. So, we investigated the effects of glial cell inhibition during the acute phase of stroke using a glial cell activation inhibitor (Ibudilast) delivered at 24 h–96 h post-ischemia. Ibudilast was chosen for its ability to reduce macrophage migration inhibitory factor (MIF) and readily cross the blood–brain barrier to rapidly suppress glial cell activation [36,37]. Following acute Ibudilast treatment, we later assessed tissue preservation (absence of astroglial scarring and macrophage recruitment) during the chronic phase of stroke (30 d), because there is a well-documented temporal relationship showing that microglial activation precedes astrocytic activation following injury [29]. In the initial 2 d–7 d post-ischemia, astrocytes change their morphology, proliferate, and migrate to the site of injury to form a scar ~14 d after the original insult [38]. Ibudilast has a half-life of 19 h [39], and its effects cease within 24 h–48 h following treatment termination. Therefore, assessments of neurogenesis and functional recovery at 30 d post-ischemia would be associated with the long-term consequences of acute inhibition of glial cell activation, rather than persistent inhibition of the neuroimmune response to stroke. 

To test the hypothesis that neurogenesis and neuroplasticity may be enhanced through neuroimmune modulation in the young brain, we compared an experimental model of pediatric ischemic stroke [22,40] in juvenile and adult mice. We examined neurogenesis with immunohistochemistry, labeling newly generated neurons and assessing colocalization with cell-type-specific markers expressed by newborn neurons throughout each stage of development [41]. We used in vivo electrophysiology to assess recovery of neuroplasticity in freely behaving mice, recording spontaneous electrical activity in the brain from depth electrodes implanted into the core of the ischemic infarct. We assessed early neuroimmune support of later neurogenesis and neuroplasticity using pharmacological inhibition of glial cell activation, immunohistochemistry, electrophysiology, and gene expression profiling. We found equivalent cellular proliferation and newly generated immature neurons in the injured brain of juveniles and adults at acute time points (7 d) post-ischemia. However, at 30 d post-ischemia, we observed substantial neurogenesis and neural regeneration only in the stroke-injured juvenile brain, with minimal brain repair detected in adults. We also found improved recovery of neuroplasticity in the stroke-injured juvenile brain at chronic time points post-ischemia (30 d), evidenced by increased electrical signaling in the ischemic core, while adult mice had persistent impairments in brain activity at all time points measured. Remarkably, inhibiting glial cell activation during the acute phase of stroke eliminated juvenile neurogenesis, tissue regeneration, and neural plasticity while rescuing neurogenesis and neuroplasticity in adults. These exciting findings not only increase our understanding of neurogenesis and post-ischemic recovery in juveniles, but also identify instrumental age-associated neuroimmune responses that support neurogenesis and neuroplasticity following stroke in juveniles which are not found in adults. 

## 2. Materials and Methods

Animals: Seventy-eight male C57BL/6 mice (Jackson Laboratory, Bar Harbor, ME, USA) were randomly assigned into groups: MCAO-injured adult, sham-operated adult, MCAO-injured juvenile, or sham-operated juvenile. Additional juvenile and adult mice were randomly assigned to the MCAO or sham groups and were administered drug or vehicle treatments. Mice were pair- or single-housed, in temperature- (23 ± 3 °C) and light- (12:12 h, light:dark) controlled rooms with ad libitum access to food and water. To reduce study attrition, particularly in the juvenile mice (due to playing or fighting) and the loss of the head cap used to collect electrophysiology data, we single-housed mice used in electrophysiology experiments. Adult and juvenile mice (MCAO and sham) were single-housed from electrode implantation throughout the duration of the electrophysiology experiments (7 d, 14 d, or 30 d post-ischemia). To reduce the effects of social isolation, we provided environmental enrichment with pre-shredded bedding material (nestlets) and housing in Bio-Huts (Bio-Serv, Flemington, NJ, USA). All procedures were performed in accordance with the Institutional Animal Care and Use Committee guidelines at Louisiana State University Health Sciences Center in Shreveport and followed the NIH Guide for the Care and Use of Laboratory Animals (8th Edition, National Research Council, 2011). 

Middle Cerebral Artery Occlusion (MCAO): The MCAO methods used were as previously reported [22,40]. Briefly, cerebral ischemia was induced under isoflurane anesthesia in juvenile (postnatal day 20–25, 10–15 g) and adult (8 weeks, 25–30 g) mice. The intraluminal monofilament model of MCA occlusion allows for a reversible, transient occlusion (45 min). We incorporated minor variations in filament size (Doccol Corporation, Sharon, MA, USA) to accommodate the smaller size of P20-25 mice. MCAO in adult and juvenile mice causes unilateral, focal cerebral ischemia, and infarct in the injured (ipsilateral) hemisphere, allowing the non-injured (contralateral) hemisphere to be used for control [22,40,42,43]. The adequacy of the MCAO was confirmed by cerebral blood flow reduction (>70% drop in cerebral blood flow required for inclusion), measured by laser Doppler flowmetry (Moor Instruments Inc., Wilmington, DE, USA) over the ipsilateral parietal cortex in all mice.

In vivo electrophysiology: Striatal depth electrodes (P1 Technologies, Roanoke, VA, USA) were implanted one week before MCAO or sham surgery under isoflurane anesthesia, as previously reported [44,45]. Electrodes were implanted at the following locations in relation to bregma: AP 0.70 mm, ML 2.0 mm, and DV 2.8 mm from the dural surface. Electroencephalography (EEG) recordings were obtained with a PowerLab acquisition system and quad bioamplifiers (ADInstruments, Colorado Springs, CO, USA). EEG signals were pre-amplified (X1000), filtered (0.1–500 Hz), and digitized (1000 Hz) and total power quantification performed with LabChart software (ADInstruments). 

Ibudilast Administration: To determine the effects of early glial cell activation in promoting neural regeneration, neurogenesis, and restoration of neuroplasticity in juvenile MCAO-injured mice compared to adult MCAO-injured mice, we attenuated glial cell activation during the acute phase of stroke (24 h–96 h post-ischemia) with Ibudilast (10 mg/kg in corn oil, s.c.; Sigma-Aldrich, St. Louis, MO, USA) or the vehicle (corn oil, s.c.). Ibudilast (3-isobutyryl-2-isopropylpyrazolo-[1, 5-a] pyridine) is a neuroimmune modulator and phosphodiesterase inhibitor that demonstrates neuroprotective, anti-inflammatory actions via glial cell inhibition [46,47,48] and antagonism of toll-like receptor 4 [49]. Ibudilast has long been used to treat patients with cerebrovascular disorders and has been found to be neuroprotective following ischemia due to its anti-inflammatory properties and anti-aggregatory actions on platelets [36,46,50,51,52,53,54]. 

Bromodeoxyuridine (BrdU) Administration: To assess neurogenesis, four injections (i.p.) of BrdU (50 mg/kg/d; Sigma-Aldrich, St. Louis, MO, USA) or vehicle (0.9% saline) were delivered at 24 h–96 h after stroke, at peak expression times reported following cerebral ischemia [55,56]. A synthetic analog of thymidine, BrdU is readily incorporated into DNA during the S-phase of the cell cycle and is a widely used and well-established reagent to label and quantify proliferating cells both in vitro and in vivo [57].

Immunofluorescence Staining: Brains were sectioned (50 μm) on a sliding microtome (Leica SM 2010 R; Leica Biosystems, Wetzlar, Germany). Free-floating sections were washed in PBS, followed by a 1 h block (5% normal donkey serum with 0.3% Triton-X), 72 h incubation at 4 °C in primary antibody, and 1 h incubation in secondary antibody. Sections were mounted with anti-fade mounting medium (Vectashield, H-1000; Vector Laboratories). Primary antibodies included the following: rat anti-BrdU (ab6326; Abcam), rabbit anti-doublecortin (ab18723; Abcam), mouse anti-NeuN (MAB377; EMD Millipore), rabbit anti-Tmem119 (ab209064; Abcam), rat anti-CD86 (ab119857; Abcam), and mouse anti-GFAP (sc-33673; Santa Cruz Biotechnology). Secondary antibodies included Alexa Fluor 488, 594, or 647-conjugated IgG (Jackson ImmunoResearch Laboratories, West Grove, PA, USA). Neurogenesis was assessed by BrdU colocalization with cell-type-specific markers at acute and chronic time points post-ischemia. For BrdU staining, sections were washed with PBS, denatured (2N HCl) for 20 min at 37 °C, and HCl was neutralized with 0.1 M borate buffer (pH 8.5). Confocal microscopy images were obtained from two sections per animal and included the injured (ipsilateral, stroked hemisphere) and non-injured (contralateral, control hemisphere) striatum using a Nikon SIM Super Resolution Microscope System (Nikon Instruments Inc., Melville, NY, USA). Fiji software was used for cell counts, percent colocalization, and pixel intensity values in the lateral striatum [58]. The cell counter plug-in and channels tool in Fiji software was used for total cell counts and percent colocalization (BrdU, DCX, and NeuN). Due to glial scarring obscuring accurate cell counts, pixel intensity values were measured for neuroimmune markers (CD86, GFAP, and TMEM119) as the mean gray value (8-bit) using the macros recorder plug-in to set the same region of interest in the lateral striatum for analyses of all images.

Gene expression analysis: Flash-frozen striatal tissue from MCAO-injured and sham-operated juvenile and adult mice were collected at 14 d post-ischemia. Total RNA was extracted using an RNeasy Mini Kit (QIAGEN©, Hilden, Germany) and treated with an RNase-Free DNase Set (QIAGEN©) to ensure DNA depletion. Total RNA across groups was pooled and analyzed as a single sample. Briefly, 1.2 μg of RNA was reverse transcribed into cDNA (60 μL) using iScript Reverse Transcription Supermix (Promega, Madison, WI, USA) according to the manufacturer’s instructions. The RT control assay template from a PrimerPCR assay (Bio-Rad, Hercules, CA, USA) was included in the master mix. Real-time PCR was performed on previously prepared cDNA, using iTaq Universal SYBR^®^ Green Supermix (Bio-Rad) in predesigned 96-well (20 μL/well) panels (PrimePCR SAB target list, cytokines, and chemokines, 10034300; Bio-Rad). PrimePCR plates were run using the CFX96 Fast Real-Time PCR System following standard qPCR protocol. Data were analyzed with PrimePCR analysis software (Bio-Rad).

Statistics: Analyses were performed by a blinded investigator. One-way ANOVAs were used to compare cell counts and colocalization of cell-type-specific markers in the ipsilateral (injured) versus contralateral (non-injured) lateral striatum of juvenile versus adult MCAO-injured or sham-operated mice at 7 d and 30 d post-ischemia. A two-way repeated measures ANOVA (Group × Time) was used to assess differences in EEG power (μV^2^) of MCAO versus sham-operated juvenile and adult mice at 7 d, 14 d, and 30 d post-injury. Additionally, two-way ANOVAs were used to assess group differences in pixel intensity values and for gene expression microarray data. Data were analyzed with GraphPad software (San Diego, CA, USA) and differences with a *p*-value of <0.05 were considered significant. All quantifications are shown as the mean  ±  SEM.

## 3. Results

### 3.1. Equivalent Neurogenesis at Acute Time Points

**Figure 1:**
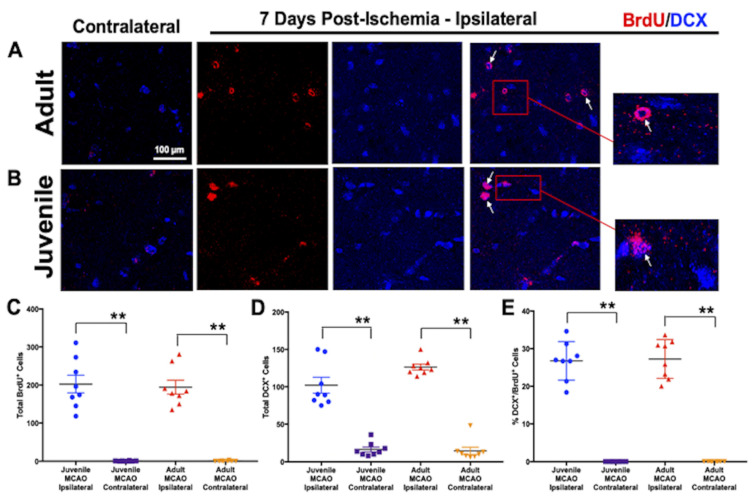
Equivalent neurogenesis at acute time points after stroke. (**A**,**B**) Representative images of BrdU^+^ (red, cell proliferation marker) and DCX^+^ (blue, immature neuron marker) expression at 60× magnification, showing similar: (**C**) proliferation of newly generated cells (BrdU^+^) and (**D**) immature neurons (DCX^+^) in the injured (ipsilateral) striatum of juvenile and adult mice at 7 d post-ischemia, compared to the uninjured (contralateral) striatum. (**E**) Increased neurogenesis was observed in both juveniles and adults in the stroke-injured hemisphere. This was evidenced by double-positive (white arrows) BrdU^+^/DCX^+^ cells (red box, zoomed inlay of newly generated immature neurons), demonstrating a marked increase in the proliferation and migration of newborn neurons to the site of injury, compared with negligible neurogenesis in the uninjured hemisphere. Data represent the mean ± SEM, *n* = 4 per group, ** *p* < 0.01.

The current studies were conducted to assess the cellular/molecular mechanisms responsible for successful neurogenesis and neuronal regeneration in the young brain, with the goal of gaining a deeper understanding of the potential for enhanced neuroplasticity and restoration of brain function in all age groups following stroke. Neurogenesis would improve network reorganization through the generation of functionally integrated neurons from progenitor cells, potentially enhancing neuroplasticity and subsequent post-stroke recovery. To examine improved neurogenesis following stroke, cerebral ischemia was transiently (45 min) induced in juvenile (postnatal day 20–25, 10–15 g) and adult (8 weeks, 25–30 g) mice, and newly generated cells were labeled with BrdU (cellular proliferation marker) at 24 h–96 h to assess neurogenesis at acute and chronic time points post-ischemia. At 7 d post-ischemia, immunostaining was performed with BrdU and doublecortin (DCX), an immature neuron marker that is almost exclusively expressed in migrating neuroblasts and immature neurons in the subventricular zone and widely used to assess adult neurogenesis [41,59]. We observed (Figure 1) a robust proliferation (F(3,28) = 59.84, *p* < 0.001) of newly generated cells (BrdU^+^, Figure 1A–C) in the injured/ipsilateral striatum that was equivalent in MCAO-injured juvenile (202.4 ± 23.2) and adult (194.3 ± 18.2, *p* = 0.9796) mice, compared to the uninjured/contralateral striatum (0.63 ± 0.26 and 0.05 ± 0.38, respectively, *p* < 0.0001). Additionally, we found increased (F(3,28) = 82.88, *p* < 0.001) numbers of immature neurons (DCX^+^, Figure 1A,B,D) that were similar in the injured striatum of MCAO-injured juvenile (102.1 ± 10.6) and adult (126.4 ± 3.9, *p* = 0.0565) mice at 7 d post-ischemia, compared to the uninjured/contralateral striatum (16.5 ± 3.3 and 14.6 ± 4.9, respectively, *p* < 0.0001). Comparative increases in neurogenesis (F(3,28) = 147.1, *p* < 0.001) were also observed in both juveniles (26.7 ± 1.8%) and adults (27.2 ± 1.8%, *p* = 0.9924) in the stroke-injured hemisphere, evidenced by increased colocalization of BrdU^+^/DCX^+^ cells (Figure 1A,B,E, white arrows), demonstrating an increased proliferation and migration of newborn neurons to the site of injury, compared with an absence of neurogenesis (0%, *p* < 0.0001 in both age groups) in the uninjured hemisphere. These findings are consistent with reports in the literature of post-stroke neurogenesis [60,61], with injury resulting in a massive proliferation and migration of newborn neurons toward the site of injury. 

### 3.2. Neuronal Regeneration in Juveniles at Chronic Time Points

**Figure 2:**
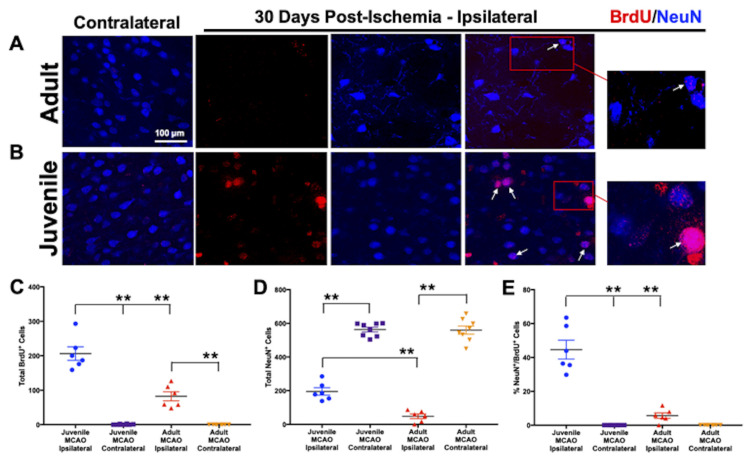
Juvenile neuronal regeneration after ischemic stroke. (**A**,**B**) Representative images of BrdU^+^ (red, cell proliferation marker) and NeuN^+^ (blue, mature neuron marker) expression at 60× magnification, showing neuronal repair and regeneration in juvenile mice, but not adults at 30 d following stroke. (**C**) Juvenile mice had persistent cellular proliferation (BrdU^+^) and (**D**) increased mature neurons (NeuN^+^) in the injured striatum compared to adult mice. (**E**) A remarkable increase in neurogenesis/neuronal regeneration was observed in the injured juvenile brain compared with adults (double-positive BrdU^+^/NeuN^+^ cells, white arrows), demonstrating that newly generated immature neurons found at 7 d went on to mature in juveniles, but few survived in the adults (red box, zoomed inlay of newborn mature neurons). Data represent the mean ± SEM, *n* = 4 per group, ** *p* < 0.01.

In stark contrast to the equivalent proliferation and migration of newly generated neurons in the injured striatum of juvenile and adult mice at 7 d, at 30 d post-ischemia, we observed a remarkable increase in neuronal regeneration in juvenile mice, but not in adults (Figure 2). We found persistently increased (F(3,24) = 91.03, *p* < 0.0001) numbers of newly generated cells (BrdU^+^, Figure 2A–C) in the injured striatum of juveniles (206.3 ± 19.5), compared to adults (82.3 ± 13.1, *p* < 0.0001). This finding is especially important in light of recent reports that striatal neuroblasts are continuously generated for months after stroke [62], indicating that continued neurogenesis and network reorganization could occur in the uniquely supportive post-ischemic microenvironment of the juvenile brain. As expected, we found few newly generated cells (BrdU^+^) in either age group in the non-injured striatum (0.75 ± 0.41 and 0.0 ± 0.0, respectively, *p* < 0.0001). We found increased (F(3,24) = 176.8 *p* < 0.001) numbers of mature neurons (NeuN^+^, Figure 2A,B,D) in the injured striatum of juveniles (195.5 ± 22.0), compared to adults (48.3 ± 13.8, *p* = 0.0002), while mature neurons were spared in the non-injured striatum due to the unilateral injury caused by MCAO (562.9 ± 13.8 and 559.8 ± 23.8, respectively, *p* < 0.0001). Incredibly, we observed marked increases (F(3,24) = 70.93, *p* < 0.001) in neurogenesis in the injured striatum of MCAO-injured juveniles (44.6 ± 5.6%), compared to adults (5.7 ± 1.6%, *p* = < 0.0001), evidenced by double-positive BrdU^+^/NeuN^+^ cells (Figure 2, white arrows). These results provide evidence that newly generated neurons migrated, matured, and replaced those lost in the injured striatum of juveniles compared to adults. Consistent with previous reports [20], adult mice had few mature newborn neurons survive.

### 3.3. Neuroimmune Support of Juvenile Neurogenesis and Neuroplasticity

**Figure 3:**
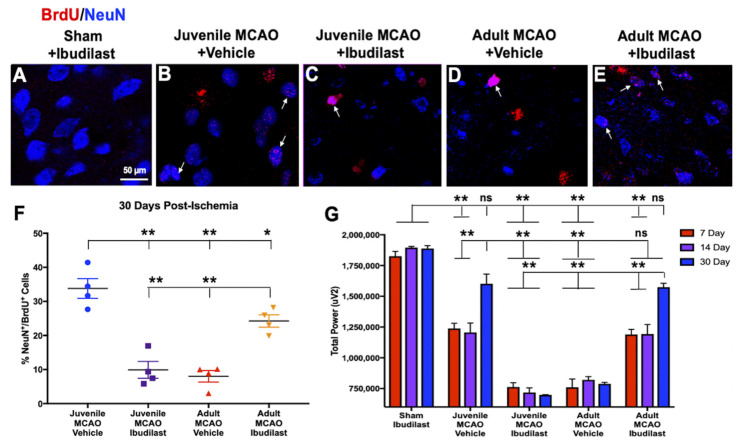
Acute neuroimmune reponses are essential for neurogenesis and neuroplasticity in juveniles. (**A**–**E**) Representative merged images (colocalization, white arrows) of BrdU^+^ (red, cell proliferation marker) and NeuN^+^ (blue, mature neuron marker) expression at 100× magnification, showing that acute glial inhibition with Ibudilast treatment (24 h–96 h) had strikingly different, long-term effects in MCAO-injured juveniles (**C**,**F**, abolished neurogenesis) versus MCAO-injured adults (**E**,**F**, rescued neurogenesis), while vehicle-treated MCAO-injured juveniles retained previously observed neurogenesis at 30 d (**B**,**F**) and minimal neurogenesis was found in vehicle-treated MCAO-injured adults (**D**,**F**). Ibudilast treatment alone did not produce any changes in neurogenesis, demonstrated by sham-operated mice (**A**). (**G**) Depth electrodes implanted in the core of the ischemic infarct to directly record the spontaneous recovery of neuronal electrical activity (total power μV^2^) in freely behaving mice demonstrated recovery of neuroplasticity in vehicle-treated MCAO-injured juveniles (**G**, 2nd columns). However, acute glial cell inhibition with Ibudilast ameliorated electrical recovery (**G**, 3rd columns) in treated juveniles, revealing essential neuroimmune support of brain plasticity after stroke in juveniles. Similar to neurogenesis findings, Ibudilast treatment in adults produced opposing effects to those found in juveniles. Inhibition of early glial cell activation restored neuroplasticity in Ibudilast-treated MCAO-injured adults (**G**, 5th columns), suggesting a detrimental role for glial cells in brain plasticity after stroke in adults, evidenced by an absence of electrical activity recovery in vehicle-treated MCAO-injured adults (**G**, 4th columns). Acute Ibudilast treatment alone did not produce any changes in electrical activity at 7 d, 14 d, or 30 d, as demonstrated by sham-operated mice (**G**, 1st columns). Vehicle-treated juveniles and Ibudilast-treated adults recovered to electrophysiological levels observed in sham-operated mice and were indistinguishable (ns) by 30 d post-ischemia. Data represent the mean ± SEM, *n* = 4 per group IHC, and *n* = 2 (sham) and *n* = 5 (MCAO) per group EEG, ** *p* < 0.01, * *p* < 0.05, ns = non-significant.

To determine if early immune system responses to stroke support juvenile neurogenesis, we inhibited glial cell activation during the acute phase of stroke (24 h–96 h) with Ibudilast (10 mg/kg, s.c.). We chose these treatment time points because it has been established in the adult literature that microglia (resident immune cells in the brain) are proinflammatory during the acute response to brain injury, which is detrimental to later neurogenesis and tissue regeneration. We wanted to examine whether the protective factors that support later newborn neuron survival, neurogenesis, and neuronal regeneration in juveniles were exerting effects during the early, acute phase of stroke. This notion was supported when we found that early inhibition of glial cell activation during the acute phase of stroke was detrimental to the neurogenesis and recovery of neuronal electrical activity in juveniles (Figure 3), demonstrating that neuroimmune responses are critical for juvenile neurogenesis and neuroplasticity after stroke. Inhibiting glial cell activation during the acute phase of stroke produced long-lasting effects, dramatically reducing (F(3,12) = 29.20, *p* < 0.0001) neurogenesis at chronic time points (30 d) post-ischemia (BrdU^+^/NeuN^+^) in the injured striatum of Ibudilast-treated, MCAO-injured juvenile mice (Figure 3C,F, 9.9 ± 2.5%), compared to vehicle-treated MCAO-injured juveniles (Figure 3B,F, 33.8 ± 2.9%, *p* < 0.0001). In adults, inhibition of glial cell activation had strikingly different effects, in which Ibudilast treatment in MCAO-injured adults increased neurogenesis (Figure 3E,F, 24.3 ± 1.8%), compared to vehicle-treated adults (Figure 3D,F, 8.0 ± 1.7%, *p* = 0.0014), revealing surprising age-related differences in early neuroimmune responses after stroke. Ibudilast treatment alone did not produce any changes in neurogenesis, as demonstrated by sham-operated mice (Figure 3A). 

Inhibiting early neuroimmune responses with acute Ibudilast treatment also revealed opposing effects on the recovery of neuroplasticity between stroke-injured juveniles and adults (Figure 3G). In vivo electrophysiology experiments directly recorded the spontaneous recovery of neuronal electrical activity at 7 d, 14 d, and 30 d after stroke from depth electrodes implanted in the core of the ischemic infarct (lateral striatum) in awake, freely behaving mice. Reductions (F(8, 27) = 5.075, *p* = 0.0006) in total power (μV^2^) were found in the injured lateral striatum at early time points (Figure 3G, 7 d and 14 d) in all MCAO-injured groups compared to Ibudilast-treated sham-operated mice (3G, 1st columns). Incredibly, at 30 d post-ischemia, we observed the recovery of neuroplasticity in the ischemic core of vehicle-treated MCAO-injured juveniles (3G, 2nd columns, 1,602,276 ± 77,858), compared to vehicle-treated MCAO-injured adults (3G, 4th columns, 788,613 ± 11,328, *p* < 0.0001). However, acute attenuation of glial cell activation with Ibudilast ameliorated neuroplasticity in MCAO-injured juveniles (3G, 3rd columns, 698,353 ± 2358, *p* < 0.0001) compared to vehicle-treated juveniles, revealing critical neuroimmune support of brain plasticity in juveniles after stroke. Similar to neurogenesis findings, Ibudilast treatment in adults produced dramatically different effects to those found in juveniles. Inhibition of glial cell activation restored neuroplasticity in Ibudilast-treated, MCAO-injured adults (3G, 5th columns, 1,573,973 ± 31,591) compared to vehicle-treated adults (3G, 4th columns, 788,613 ± 11,328, *p* < 0.0001), suggesting a detrimental role for early glial cell activation in brain plasticity in adults after stroke. Additionally, adult Ibudilast-treated mice did not differ from vehicle-treated juveniles at any time point post-ischemia (*p* > 0.9999), and the same was true for Ibudilast-treated juveniles compared to vehicle-treated adults (*p* > 0.9999). This finding revealed an inverse relationship in post-ischemic neuroimmune responses in MCAO-injured juveniles compared to adults, demonstrating important age-related differences in neuroimmune signaling during the acute phase of stroke. These results also provide further evidence for the hypothesis that acute post-ischemic glial cell activation contributes essential support for the juvenile neurogenesis and recovery of neuroplasticity, while causing damage in adults. Treatment with Ibudilast alone did not produce any changes in electrical activity at 7 d, 14 d, or 30 d, as demonstrated by sham-operated mice (3G, 1st columns). Surprisingly, both vehicle-treated MCAO-injured juveniles and Ibudilast-treated MCAO-injured adults recovered to electrophysiological levels observed in sham-operated mice and were indistinguishable (*p* = 0.2289 and *p* = 0.0569, respectively) by 30 d post-ischemia.

### 3.4. Age-Associated Neuroimmune Responses Promote Neuroprotective Tissue Preservation after Juvenile Stroke 

**Figure 4:**
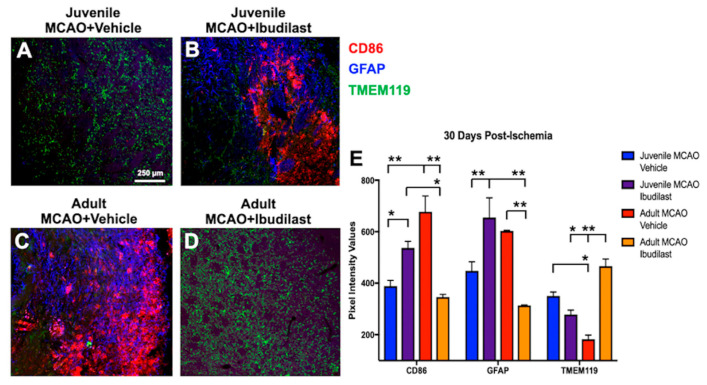
Stroke-induced neuroimmune responses promote tissue preservation and reduced glial scarring in juveniles. (**A**–**D**) Representative merged images of CD86^+^ (red, macrophage/M1 marker), GFAP^+^ (blue, astrocyte marker), and TMEM119^+^ (green, microglia-specific marker) immunoreactivity at 20× magnification, showing the following: (**B**) acute attenuation of glial cell activation with Ibudilast in MCAO-injured juveniles reversed tissue preservation seen in vehicle-treated MCAO-injured juveniles (**A**), increasing glial scarring, astrogliosis, and proinflammatory macrophage expression. In sharp contrast, Ibudilast treatment in MCAO-injured adults resulted in a dramatically reduced proinflammatory response and increased tissue preservation at 30 d (**D**) that was similar to vehicle-treated juveniles (**A**). Additionally, vehicle-treated adults (**C**) were similar to Ibudilast-treated juveniles (**B**) with increased glial scarring, diffuse astrogliosis, and proinflammatory macrophage immunoreactivity in the infarcted striatal core. (**E**) Quantification of pixel intensity values revealed a sharp increase in proinflammatory macrophages (CD86^+^) and astrocytes (GFAP^+^) in Ibudilast-treated juvenile and vehicle-treated adult mice, supporting opposing neuroimmune responses that are protective in juveniles and damaging in the adult brain acutely following stroke. Interestingly, microglial (TMEM119^+^) intensity values were increased in MCAO-injured mice with tissue preservation, both vehicle-treated juveniles, and Ibudilast-treated adults. However, their morphology appears to be ramified, rather than reactive. Further, tissue preservation resulted in greater overall cell survival compared to mice with massive glial scarring, and a reduction in infiltrating macrophages and astrogliosis is more indicative of a reduced proinflammatory state. Data represent the mean ± SEM, *n* = 3 per group, ** *p* < 0.01, * *p* < 0.05.

We observed that acute inhibition of stroke-induced glial cell activation (24 h–96 h post-ischemia) with Ibudilast treatment resulted in neurodegenerative tissue damage in juveniles at 30 d post-ischemia (Figure 4). Tissue preservation previously found in vehicle-treated MCAO-injured juveniles (Figure 4A) was lost at 30 d following short-term attenuation of glial cell activation in Ibudilast-treated MCAO-injured juvenile (Figure 4B) mice, evidenced by increased proinflammatory macrophage immunoreactivity (CD86^+^, activated microglia/macrophage and M1 marker), diffuse astrogliosis (GFAP^+^, astrocyte marker), and glial scarring in the lateral striatum at 30 d post-injury. These results suggest that glial cells are neuroprotective during the acute phase of juvenile stroke and play a role in extended tissue preservation. In sharp contrast, we found that Ibudilast treatment in MCAO-injured adults resulted in a dramatically reduced proinflammatory response and remarkably improved tissue preservation (Figure 4D), compared with vehicle-treated MCAO-injured adults (Figure 4C), again revealing differing age-related immunomodulatory effects in juvenile versus adult mice after stroke.

### 3.5. Proinflammatory Mediators following Stroke

Quantification of pixel intensity values revealed increases (F(6,24) = 12.24, *p* < 0.0001) in proinflammatory macrophage (CD86^+^) intensity values in Ibudilast-treated juveniles, compared to vehicle-treated juveniles (Figure 4E, 536.6 ± 26.2 and 388.3 ± 22.5, respectively, *p* = 0.0275) and Ibudilast-treated adult mice (Figure 4E, 346.0 ± 10.6, *p* = 0.0196), demonstrating that early attenuation of glial cell activation resulted in more proinflammatory macrophage expression compared to juveniles without attenuation (vehicle) and adults with attenuation (Ibudilast). The same was found for vehicle-treated adults (Figure 4E, 676.9 ± 61.4), in which we observed increased proinflammatory macrophage (CD86^+^ ) intensity values compared to juveniles without the inhibition of glial cell activation (vehicle-treated, Figure 4E, 388.3 ± 22.5, *p* = 0.0004) and adults with glial cell inhibition (Ibudilast-treated, Figure 4E, 346.0 ± 10.6, *p* = 0.0004). These results showed that attenuating glial cell activation in juveniles or not attenuating in adults produced similar proinflammatory responses, again showing support for a neuroprotective role for early glial cell activation in tissue preservation in juveniles and in a neurodegenerative role in adults. When glial cells remained activated in vehicle-treated MCAO-injured juveniles, or when activation was inhibited in Ibudilast-treated adults, we observe reductions in CD86^+^ (Figure 4E, 388.3 ± 22.5 and 346.0 ± 10.6, respectively, *p* = 0.8938) intensity values. 

The same pattern was found for GFAP^+^ intensity values in Ibudilast-treated juveniles, compared to vehicle-treated juveniles (Figure 4E, 655.1 ± 76.2 and 447.7 ± 35.2, respectively, *p* = 0.0015) and Ibudilast-treated adult mice (Figure 4E, 313.4 ± 1.5, *p* < 0.0001), demonstrating increased GFAP^+^ intensity values compared to vehicle-treated juveniles and Ibudilast-treated adults. The same was found for vehicle-treated adults (Figure 4E, 602.9 ± 2.7), in which we observed increased GFAP^+^ intensity values compared to vehicle-treated juveniles (Figure 4E, 447.7 ± 35.2, ns *p* = 0.0711) and Ibudilast-treated adults (Figure 4E, 313.4 ± 1.5, *p* = 0.0017). These results demonstrated that inhibiting glial cell activation in juveniles or not inhibiting glial cell activation in adults produced similar astrocytic responses and increased glial scarring, providing more evidence for opposing neuroimmune responses that are protective in juveniles and damaging in the adult brain following stroke. When glial cells remained activated in vehicle-treated MCAO-injured juveniles and inhibited in Ibudilast-treated MCAO-injured adults, we observed reductions in GFAP^+^ (Figure 4E, 447.7 ± 35.2 and 313.4 ± 1.5, respectively, *p* = 0.1402) intensity values. 

Interestingly, microglial (TMEM119^+^, microglia-specific marker, green) intensity values were increased in vehicle-treated juveniles, compared to vehicle-treated adults (Figure 4E, 349.7 ± 15.7 and 181.9 ± 16.1, respectively, *p* = 0.0456). Additionally, Ibudilast-treated adults had higher intensity values compared to Ibudilast-treated juveniles (Figure 4E, 466.0 ± 27.3 and 278.6 ± 16.3, respectively, *p* = 0.0221) and vehicle-treated adult mice (Figure 4E, 181.9 ± 16.1, respectively, *p* = 0.0021). However, their microglial morphology appears to be more ramified, rather than reactive. Further, tissue preservation resulted in higher overall cell survival compared to mice with massive glial scarring, and a reduction in infiltrating macrophages and astrogliosis is more indicative of a reduced proinflammatory state. 

### 3.6. Anti-Inflammatory Mediators following Stroke

**Figure 5:**
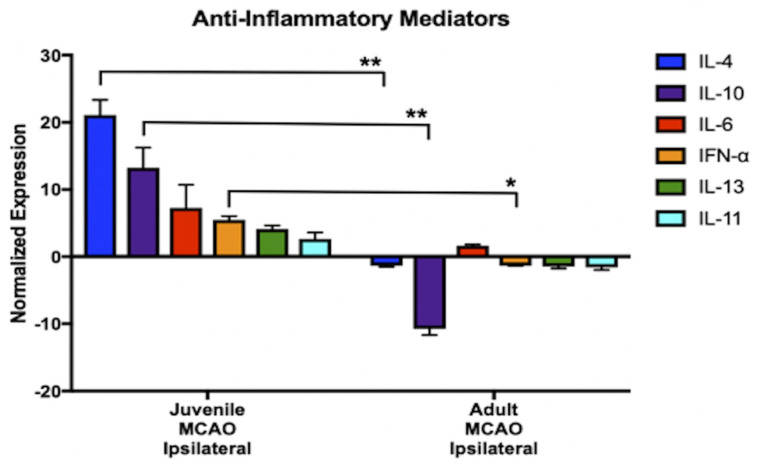
Anti-inflammatory mediators following stroke. Gene expression profiles were assessed at 14 d following stroke to target the subacute phase of stroke during the time that immature neurons begin to mature, to gain better insight into protective mediators in the neurogenic environment. The analysis revealed differential expression in many of the major anti-inflammatory mediators reported after stroke, which were upregulated in MCAO-injured juveniles and downregulated in MCAO-injured adults, revealing that the injured striatum of juveniles was supported by a predominantly anti-inflammatory gene expression profile compared to MCAO-injured adults. The highest expression was in the two cytokines Interleukin 4 (IL-4, an anti-inflammatory phenotype inducer that drives microglia/macrophages toward an anti-inflammatory signature) and Interleukin 10 (IL-10, an anti-inflammatory cytokine important for immunoregulation), which has also been reported to promote neural progenitor cell differentiation and neurogenesis. Data represent the mean ± SEM, *n* = 6 per group, ** *p* < 0.01, * *p* < 0.05.

Post-ischemic glial cell activation sets forth a cascade of events that modulate neurogenesis through the secretion of different soluble factors (e.g., cytokines, chemokines, trophic factors, etc.) that affect the proliferation, differentiation, and survival of newborn neurons [23] as well as axonal and dendritic growth, synapse formation, and synaptic plasticity [24]. We performed exploratory gene expression profiling analyses of cytokines and chemokines at 14 d post-ischemia (Figure 5) to identify possible targets for the neuroimmune support of juvenile neurogenesis and recovery of neuroplasticity during the time that immature neurons begin to mature. This time point was chosen in order to gain better insight into the instrumental neuroimmune signaling pathways that position the juvenile brain for better post-ischemic recovery compared to adults. The analysis revealed differential expression (normalized to 18S rRNA) in many of the major anti-inflammatory mediators reported after stroke, which were all upregulated in MCAO-injured juveniles and downregulated in MCAO-injured adults, demonstrating that the injured striatum of juveniles is supported by a predominantly anti-inflammatory gene expression profile compared to MCAO-injured adults. This is also the time shortly before newborn neurons mature and subsequent neuroplasticity is restored, and these age-associated differences in anti-inflammatory signaling could explain the differences found in post-ischemic recovery in juveniles compared to adults. The highest expression was found in two secreted cytokines: Interleukin 4 (IL-4, an anti-inflammatory phenotype inducer that has been shown to support neurogenesis and tissue neuroprotection) and Interleukin 10 (IL-10, an anti-inflammatory cytokine that has been reported to promote neural progenitor cell differentiation and neurogenesis). 

## 4. Discussion

Pediatric stroke is understudied, and there are few reports of the response to cerebral ischemia in the developing brain. The current results not only increase our understanding of endogenous neural regeneration and post-ischemic recovery of neuroplasticity, but also identify an essential role for neuroimmune responses in supporting juvenile neurogenesis and enhanced functional recovery after stroke. To our knowledge, this study is the first to report the neuroimmune support of neurogenesis and neuroplasticity following pediatric stroke. The ability to study neurogenesis in the uniquely supportive microenvironment of the juvenile brain provides new opportunities to identify targets that are also relevant to adult and aging populations. The key findings from these experiments were the following: (1) at 7 d following stroke, equivalent neurogenesis (colocalization of BrdU^+^/DCX^+^ cells) was observed in the damaged striatum of both MCAO-injured juvenile and adult mice; (2) yet, at 30 d post-ischemia, we discovered a marked increase in neurogenesis (colocalization of BrdU^+^/NeuN^+^ cells) only in the injured striatum of juveniles, compared with the minimal survival of newly generated neurons in adults; (3) we found increased neuroplasticity at 30 d post-ischemia in juvenile mice, while adults exhibited persistent deficits in brain activity; (4) remarkably, inhibiting glial cell activation during the acute phase of stroke abolished juvenile neurogenesis and neuroplasticity and rescued neurogenesis and neuroplasticity in adults; (5) neuroglia were found to be critical for reduced proinflammatory responses and tissue preservation after stroke in juveniles, evidenced by increased proinflammatory immunoreactivity and glial scarring following acute inhibition of glial cell activation; (6) post-ischemic attenuation of glial cell activation was found to be beneficial in the adult brain, revealing opposing effects to those found in juveniles. Acute inhibition of glial cell activation in adults reduced the expression of proinflammatory markers and glial scarring, and rescued tissue preservation. (7) Gene expression profiling analyses revealed that anti-inflammatory gene expression was upregulated in juveniles and downregulated in adults during the time shortly before functional recovery of neuroplasticity, which was also during the time that immature neurons begin to differentiate into mature neurons. These observations provided our first insight into essential immune mediators that support the post-ischemic juvenile brain and identified an important role for neuroimmune mechanisms in age-related differences found in neurogenesis and functional recovery. 

Neuronal damage and death in brain regions injured by stroke contribute to long-term disability, and endogenous neurogenesis has the potential to replenish damaged neurons and restore functioning to the injured brain. Neurogenesis occurs in the brain of both juveniles and adults. However, it is well established that in adults, these cells quickly die without contributing to post-ischemic recovery and lack therapeutic potential [19,61,63]. Further, there are low survival rates in implanted neural stem cells following stroke in adults [64,65]. Increased understanding of the conditions needed to support neuronal maturation in the stroked brain could help inform the rapidly growing field of stem cell therapy, and new reports in the literature suggest that pre-treated stem cells and/or anti-inflammatory therapies in stroke patients may hold promise for future interventions [66]. In the pediatric population, even less is known about the mechanisms underlying recovery after stroke. Unfortunately, due to the lack of universal guidelines for childhood stroke, many therapies used in adults are used for pediatric stroke management [13,67]. Numerous clinical trials in adults have focused on reducing proinflammatory signaling following stroke [30,68,69], and our results suggest that these therapies could have devastating consequences in the pediatric population. Future research is crucial in this poorly studied population in order to establish therapies for pediatric stroke survivors. The current findings reveal that the juvenile brain is very different from adults, and treatments used in adults could hinder functional recovery in pediatric stroke survivors. 

We observed increased juvenile neurogenesis at acute (7 d) and chronic (30 d) time points post-ischemia (Figure 1 and Figure 2), which demonstrated that the microenvironment in the young brain was uniquely supportive of neurogenesis and neural regeneration in stroke-damaged brain regions at chronic time points, whereas the adult brain was not. We have previously reported that the neurogenesis and neuronal repopulation of the injured juvenile striatum is coupled with improved motor outcomes after ischemic stroke [22]. We also characterized striatal neuron subtypes in juveniles and found that newly generated mature neurons replaced region-specific medium spiny neurons in the injured striatum. The present results validate and extend those findings, demonstrating enhanced neuroplasticity in juveniles, which likely contributed to improved post-ischemic motor outcomes. In addition to neuronal regeneration, the study outcomes provided compelling evidence for improved post-ischemic recovery of neuroplasticity (Figure 3) in juveniles, but not in adults, highlighting the role of neurogenesis in improved brain plasticity after stroke. Reorganization in stroke-damaged brain regions is known to improve stroke recovery [14,15], and newly generated neurons may integrate into stroke-damaged neural networks and subsequently improve post-stroke recovery. Investigating neuroplasticity deficits and recovery in preclinical animal models of stroke are critical for developing translational applications that promote improved neuroplasticity following stroke. Electroencephalography is a non-invasive method that has been used to diagnose and monitor human ischemic stroke [70], and it is also closely related to cerebral blood flow (CBF). When CBF drops, faster EEG frequencies are lost and slower frequencies (i.e., delta) increase, representing a critical ischemic threshold for neuronal cell death [71]. More widespread clinical use of EEGs in stroke patients would improve translational preclinical trials, potentially providing important diagnostic–therapeutic algorithms in human stroke patients that better predict neuronal cell death and long-term functional outcomes. In contrast to the adult literature, the present findings support the development of new therapies that target neurogenesis for stroke rehabilitation to improve the restoration of brain function and reduce stroke-related disabilities. At early time points post-ischemia (7 d), neurogenesis and impaired brain activity were equivalent in juveniles and adults, suggesting that the therapeutic window for post-ischemic regeneration and recovery may be longer than previously thought. Determining the mechanisms responsible for successful neural repair and regeneration in the young brain could provide a deeper understanding of the plasticity potential in the stroke-injured brain and opportunities for the advancement of novel stroke therapies that stimulate neurogenesis and the restoration of neuroplasticity in pediatric, adult, and elderly populations. 

We investigated stroke-induced glial cell activation as a possible mechanism underlying juvenile neurogenesis because it is well established that glial cells are involved at every stage of development in newly generated neurons, from proliferation to synapse formation. Stroke triggers the activation of microglia, which are the primary antigen-presenting cells in the CNS and first responders to mount the innate immune response to stroke and brain injury [30,72]. Once activated, microglia quickly polarize into proinflammatory and/or anti-inflammatory phenotypes [73]. In adults, it has been established that neuroglia are classically activated (proinflammatory) acutely following stroke, but we found that the acute neuroimmune response to stroke in juveniles is primarily anti-inflammatory. We investigated the neuroimmune support of juvenile neurogenesis through the inhibition of glial cell activation during the acute phase of stroke (24 h–96 h), during the timeline reported in the adult literature to be proinflammatory and detrimental to neurogenesis and tissue regeneration. On the contrary, we found that protective factors supporting later newborn neuron survival and neuronal regeneration were exerting effects during the early, acute phase of stroke (Figure 3 and Figure 4). We found that early attenuation of glial cell activation during the acute phase of stroke produced damaging neuronal effects in juveniles, eliminating neurogenesis and recovery of neuroplasticity at chronic time points (30 d) post-ischemia. Acute attenuation of glial cell activation also resulted in neurodegenerative tissue damage in juveniles at 30 d post-ischemia, where previously observed tissue preservation (Figure 4A) was lost and replaced with massive glial scarring (Figure 4B), indicating that early neuroimmune signaling is vital for extended tissue preservation after juvenile stroke. Opposing effects were found in adults, where acute attenuation of glial cell activation dramatically reduced proinflammatory signaling and astroglial scarring, while increasing tissue preservation (Figure 4D) in Ibudilast-treated MCAO-injured adults, compared to vehicle-treated adults (Figure 4C). Findings in adults were consistent with reports in the literature indicating that microglia are involved in the onset and maintenance of astrogliosis during classical, proinflammatory activation states following brain injury [29]. However, it should be noted that both microglia and astrocytes are involved in the acute neuroimmune response to stroke, and both play an important role in the growth and differentiation of newly generated neurons through the release of growth factors and energy supplies [74]. Molecules produced by astrocytes can influence the survival and differentiation of adult neural progenitor cells [28]. Ibudilast treatment also inhibits astrocytes, which may be involved in the loss of neurogenesis in juveniles and rescue in adults. Like astrocytes, oligodendrocytes are inhibited by Ibudilast [48] and have also been found to play an important role in neuronal survival and neuroplasticity [75]. So, it cannot be ruled out that the inhibition of oligodendrocytes could also be involved in the loss of juvenile neurogenesis and neuroplasticity and restoration in adults. Although it is beyond the scope of the current study, we plan to characterize the effects of augmentation and inhibition of microglia, astrocytes, oligodendrocytes, and neurons during the acute, subacute, and chronic phases of stroke to further examine age-associated differences in the post-ischemic cellular environment. 

In the present study, we discovered opposing age-associated neuroimmune responses during the acute phase of stroke, which has far-reaching implications for stroke management in both pediatric and adult populations. While other investigators have found beneficial effects of neuroimmune responses after stroke in adults, it is typically during the chronic phase of stroke [30,74,76], after irreparable damage to newly generated neurons has already occurred. Instead, we observe a predominantly anti-inflammatory gene expression profile in MCAO-injured juveniles during the time shortly before newborn neurons matured and subsequent neuroplasticity was restored, while expression of all anti-inflammatory mediators was downregulated in adults following stroke (Figure 5). Neurogenesis is modulated through the secretion of cytokines following a CNS insult, which act upon glia, neurons, circulating immune cells, and the cerebrovascular endothelium [23,77,78]. We found increased gene expression of two potent anti-inflammatory cytokines, IL-4 and IL-10, which may be involved in neuroimmune signaling pathways supporting juvenile neurogenesis and functional recovery. IL-4 is a STAT6 activator that shifts microglia/macrophages toward an anti-inflammatory phenotype following stroke [79,80] and has been reported to promote neurogenesis, tissue protection, and repair [81,82]. Signaling from IL-10 pathways could also improve juvenile neurogenesis and functional recovery, since IL-10 exerts important immunomodulatory effects on proinflammatory cytokine and chemokine production [83] and also promotes neural stem/progenitor cell differentiation and neurogenesis [81]. Taken together, these findings provide evidence that early neuroimmune responses and anti-inflammatory signaling contribute to enhanced juvenile neurogenesis and neuroplasticity after stroke. In contrast, early neuroimmune responses to adult stroke were proinflammatory and failed to support the survival of newly generated neurons at 30 d post-injury. Acute inhibition of stroke-induced glial cell activation did rescue neurogenesis and neuroplasticity in adults, supporting multiple reports of proinflammatory neuroimmune responses during the acute phase of stroke in adults. However, acutely blocking proinflammatory signaling cascades in adult stroke is an unlikely therapeutic target due to complex interactions between immune cells and other cell types, in both the CNS and PNS. Glial cells also have diverse functions and alteration in functionality could produce many off-target treatment effects. Yet, new immune therapies aim to neutralize pathogenic signaling and neuroimmune modulation is a key target in the development of new stroke therapies [68,84]. The pediatric stroke model used in the current report will be used in future studies to expand on these promising findings, distinguishing the types of immune cells involved and identifying essential cellular signaling pathways that promote regeneration and recovery in the young brain. 

## 5. Conclusions

Overall, we found that the juvenile microenvironment is uniquely supportive of neurogenesis, neuroplasticity, and tissue preservation. We observed age-related differences in neurogenesis and neuroimmune responses that are quite different from reports in the literature. We report here a critical window for glial cell activation that affects later neuronal regeneration and functional recovery at 30 days in both juveniles and adults. In adults, it has been well established that proinflammatory neuroimmune responses are more commonly found during the acute response to brain injury, which is detrimental to neurogenesis and tissue regeneration [61,63,85]. However, by acutely following juvenile stroke, we found support for anti-inflammatory signaling (Figure 3, Figure 4 and Figure 5), and acute attenuation of glial activation resulted in the loss of neurogenesis, neuroplasticity, and tissue preservation. Surprisingly, the same critical window for glial cell inhibition to support neural regeneration and recovery in adults led to the loss of regeneration and recovery in juveniles. These findings shed light on neuroimmune mechanisms underlying neurogenesis and functional recovery in juveniles and suggest the possibility that the early inhibition of microglial activation during the acute phase of stroke may be a promising target for improving the survival of endogenous newborn neurons and post-ischemic functional recovery in adults. Further, enhancing anti-inflammatory signaling, possibly in combination with stem cell therapy, could also show promise in improving neural regeneration and neuroplasticity in pediatric, adult, and aging populations. 

## Data Availability

All data presented in this study are included in the Materials and Methods section, in the corresponding References section, and are available upon reasonable request from the corresponding author (krista.rodgers@lsuhs.edu).
